# DNA copy number analysis of metastatic urothelial carcinoma with comparison to primary tumors

**DOI:** 10.1186/s12885-015-1192-2

**Published:** 2015-04-09

**Authors:** Richard M Bambury, Ami S Bhatt, Markus Riester, Chandra Sekhar Pedamallu, Fujiko Duke, Joaquim Bellmunt, Edward C Stack, Lillian Werner, Rachel Park, Gopa Iyer, Massimo Loda, Philip W Kantoff, Franziska Michor, Matthew Meyerson, Jonathan E Rosenberg

**Affiliations:** 1 Memorial Sloan Kettering Cancer Center/Weill Cornell Medical College, New York, USA; 2 Dana-Farber Cancer Institute/Harvard Medical School, Boston, MA USA; 3 The Broad Institute of MIT and Harvard, Cambridge, MA USA

## Abstract

**Background:**

To date, there have been no reports characterizing the genome-wide somatic DNA chromosomal copy-number alteration landscape in metastatic urothelial carcinoma. We sought to characterize the DNA copy-number profile in a cohort of metastatic samples and compare them to a cohort of primary urothelial carcinoma samples in order to identify changes that are associated with progression from primary to metastatic disease.

**Methods:**

Using molecular inversion probe array analysis we compared genome-wide chromosomal copy-number alterations between 30 metastatic and 29 primary UC samples. Whole transcriptome RNA-Seq analysis was also performed in primary and matched metastatic samples which was available for 9 patients.

**Results:**

Based on a focused analysis of 32 genes in which alterations may be clinically actionable, there were significantly more amplifications/deletions in metastases (8.6% vs 4.5%, p < 0.001). In particular, there was a higher frequency of *E2F3* amplification in metastases (30% vs 7%, p = 0.046). Paired primary and metastatic tissue was available for 11 patients and 3 of these had amplifications of potential clinical relevance in metastases that were not in the primary tumor including *ERBB2*, *CDK4, CCND1*, *E2F3,* and *AKT1*. The transcriptional activity of these amplifications was supported by RNA expression data.

**Conclusions:**

The discordance in alterations between primary and metastatic tissue may be of clinical relevance in the era of genomically directed precision cancer medicine.

**Electronic supplementary material:**

The online version of this article (doi:10.1186/s12885-015-1192-2) contains supplementary material, which is available to authorized users.

## Background

Bladder cancer is diagnosed in approximately 400,000 people and causes 150,000 deaths worldwide each year [1]. The majority of urinary tract cancers in the developed world are of urothelial carcinoma (UC) histology [2]. Extensive data characterizing the genetic profile of primary UC has been published and includes The Cancer Genome Atlas (TCGA) project which comprehensively describes the molecular features of primary muscle-invasive bladder UC [3]. These studies have identified several recurrent and therapeutically targetable genetic alterations but have focused on primary tumor characterization rather than the metastatic lesions that ultimately cause patient death. In muscle-invasive UC, these alterations include somatic point mutations in *TP53* (35-50%), *PIK3CA* (15-20%) and *FGFR3* (10-15%) [3-5]. Inactivating mutations commonly occur in chromatin remodeling genes, most frequently *MLL2, ARID1A* and *KDM6A,* each of which occur in approximately 25% of cases [3,6]. Furthermore, oncogenic somatic copy-number alterations (SCNAs) have been described including deletion of *RB1* in 14-15% and amplification of *ERBB2* in 5-7% of cases [3,5]. Copy number loss in chromosome 9 and copy number gain in the q arm of chromosome 8 are common, although their exact biologic significance is uncertain [7,8]. Previous studies have shown that *FGFR3* and *KDM6A* mutations are associated with lower grade and stage primary tumors, while *RB1* deletion and *TP53* mutations are more common in high-grade tumors [4,6-8]. One study used next-generation sequencing to examine alterations in 182 cancer-related genes in a cohort of 35 locally advanced or metastatic UC patients [9]. The majority of samples analyzed were from the primary tumor and results were broadly similar to what was previously reported in muscle-invasive primary bladder UC cohorts [9]. In this study, we sought to characterize the genome-wide SCNA profile in a cohort of metastatic UC samples. Furthermore, we compared these metastases to primary tumors using SCNA and RNA expression analysis to understand the genetic and transcriptomic differences between these two disease states and to identify changes associated with progression from primary to metastatic disease.

## Methods

Details on the site of tumor tissue, normal tissue, age and gender for the 46 individual cases analyzed are outlined in Table 1 and Additional file 1: Table S1. 30 metastatic UC samples were analyzed from lung, node and other sites. These were compared with 29 primary UC samples mostly of bladder origin. Paired primary and metastatic tissue was available for the same patient in 11 cases. RNA data was available in 9 of these 11 matched pairs.

**Table 1:** **Sites of primary tumour and metastases analysed**

	n	%
**Primary tumors (n=29)**		
Bladder	24	83%
Upper tract	5	17%
**Metastases (n=30)**		
Lung	10	33%
Peritoneum	6	20%
Lymph node	6	20%
Brain	3	10%
Other	5	17%

Details of individual cases are outlined in Additional file 1: Table S1.

Following pathologic examination, tumor DNA was extracted from formalin fixed paraffin embedded (FFPE) tissue using the QIAamp DNA FFPE Tissue Kit (Qiagen, Valencia, CA) as previously described [10]. Where available, normal DNA for comparison was extracted from adjacent histopathologically normal lymph nodes, renal parenchyma, seminal vesicle, prostate or lung tissue. Using the same samples, total RNA was extracted when possible using the automated Beckman Coulter Biomek FxP platform and the Agencourt Formapure Kit.

Copy number analysis for normal, primary tumor and metastatic DNA was performed using MIP array technology (Affymetrix OncoScan FFPE Express 2.0) with 334,183 sequence tag site probes which were used to measure DNA copy number at different loci across the human genome [11]. Probes were spaced at a median of 9 kb between each locus but were distributed closer together at known oncogenes and tumor suppressor genes. Copy number data were processed and normalized by Affymetrix as previously described [11]. Copy numbers were estimated with the NEXUS software and only samples that passed Affymetrix quality control metrics (median absolute pairwise difference [MAPD] value of ≤ 0.6) were considered [12].

Two micrograms of total RNA from each sample was utilized for sequencing library construction. Complementary DNA (cDNA) synthesis and bar-coded sequencing library preparation was performed as previously described [13,14] with the following modifications: Double-stranded cDNA synthesis was performed using random hexamers and cDNA was purified using QiagenTM mini-elute columns. Samples were mixed (six samples per lane of Illumina V3 HiSeq sequencing) and 101 base pair paired-end sequencing was performed. The resultant data was aligned to the human reference genome (hg19) and exon-exon junctions (ensembl v64) with the PRADA pipeline [15]. Non-human sequences were taxonomically characterized using PathSeq, as previously described [16]. Gene-level expression values [in reads per kilobase per million mapped reads (RPKM)] were generated by RNA-Seq for transcriptomic analysis [17].

The frequency of SCNA across the whole genome was assessed to compare alteration frequencies between primary tumors and metastases. A focused analysis was also performed to look for amplifications/deletions in genes involved in proliferation and cell-cycle control known to commonly harbor oncogenic alterations in UC and for which targeted therapies are currently under investigation [3,5]. This focused analysis also examined the frequency of amplifications/deletions in regions found to have statistically significant focal SCNAs using the Genomic Identification of Significant Targets in Cancer version 2.0 algorithm (GISTIC2.0) in the TCGA analysis [3].

There are no standardised log_2_ ratio cut-offs to define low-amplitude copy number gain/loss and high amplitude amplification/deletion. Based on the available published literature, we used a log_2_ ratio cut-off of +/− 0.25 to define copy number gain/loss and a log_2_ ratio cut-off of +/− 0.8 to define amplification and deletion [7,18,19].

Normalized copy number data was segmented using GLAD with default parameters available in GenePattern version 3.3.3 [20]. GISTIC 2.0 (v2.0.12) was then used to identify regions of the genome that were significantly gained or deleted across a set of samples using a Q-value cutoff <0.25 [21]. This algorithm is designed to identify significant driver SCNAs in human cancers by taking into account the frequency and amplitude of the SCNA and comparing it to the background rate of SCNAs across the genome. The algorithm compensates for the different background frequencies of SCNAs of varying length and quantifies the likelihood of copy-number alterations being biologically relevant in the form of a q-value. The software estimated false discovery rates (q-values), as well as potential targets (drivers) within the copy number aberrant regions. Threshold for copy number gain and loss was set at +/− 0.25 so that approximately 99% of all segments in normal samples were below this threshold. We defined broad alterations as those spanning >50% of a chromosome arm.

To infer the relative similarity between the DNA and RNA profiles of normal, primary and metastatic samples, unsupervised hierarchical clustering was performed as follows: for the DNA data, hierarchical clustering was performed using the pvclust R package with 1000 bootstrap iterations, Ward’s clustering method and otherwise default parameters. The boot strapping procedure estimates how strongly the clusters are supported by data. Bootstrap values are reported as percentages and indicate how often a cluster was observed in the bootstrapping. They are obtained by multiscale [22,23] and by normal resampling, i.e. sampling with replacement.

For RNA data, unsupervised hierarchical clustering was performed and RNA-Seq RPKM values were log2 + 1 transformed. Invariantly expressed genes were removed using the genefilter R package. Using the default settings of this package, we removed 50% of the genes with lowest interquartile range (IQR). Clustering was then performed with the same parameters we used for the DNA data.

To further test for the clonality of matched primary tumors and metastases, the Clonality testing R package tool developed at Memorial Sloan Kettering Cancer Center was used to analyze the DNA copy number data [22-24]. This is an R package for testing whether two tumors from the same patient are clonal (metastasis) or independent (synchronous primaries) based on their genome wide copy number profiles.

For the RNA data, heatmaps and tables of differentially expressed genes in normal bladder vs. primary and metastases and in primary vs. metastases are presented (Additional file 2: Figure S1 and Additional file 3: Figure S2).

All samples were collected under protocols approved by the Institutional Review Board (IRB) at Dana Farber Cancer Institute, de-identified and approved for use by the DFCI IRB.

## Results

**Figure 1:**
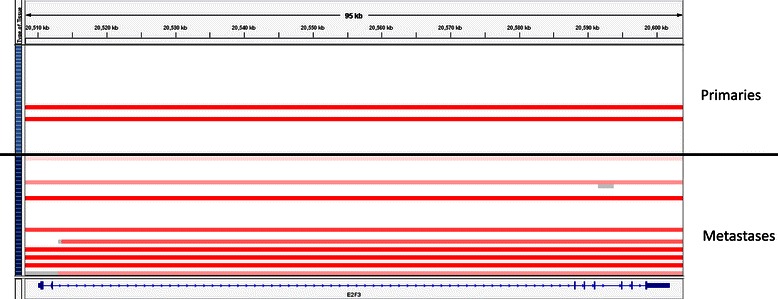
***E2F3*amplification in primary tumors vs. metastases.** Analysis of *E2F3* gene copy number data using IGV with each row representing a single tumor sample. Primary tumor samples are arrayed above the black line and metastases below it. On the left side of the diagram, the light blue boxes represent primary tumor samples and the dark blue boxes represent metastases. Red bars represent amplification (log_2_ copy number ratio >0.8).

**Figure 2:**
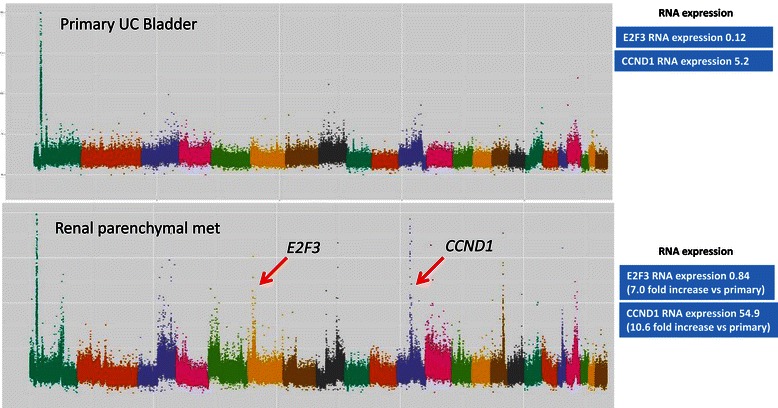
**DNA copy number and RNA expression data from patient #25.** Copy number plot with the x-axis denoting each point across the genome - each chromosome is highlighted in a different colour starting with chromosome 1 on the left side. Y-axis enumerates the log-2 copy number value at each point across the genome.Red arrows indicate gene amplifications highlighted in this manuscript and corresponding RNASeq expression readouts are displayed in blue boxes. Normal *E2F3* copy number in primary tumour (log_2_ copy number ratio 0.10) and amplification of *E2F3* in the metastasis (log_2_ copy number ratio 0.85). Normal *CCND1* copy number in primary tumour (log_2_ copy number ratio 0.60) and amplification of *CCND1* in the metastasis (log_2_ copy number ratio 3.29).

**Figure 3:**
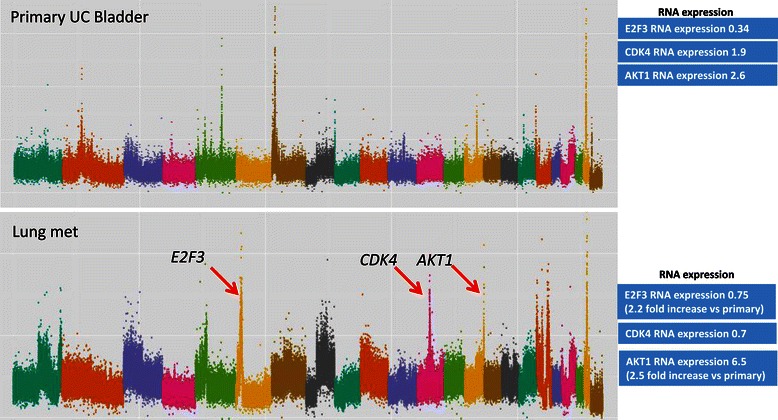
**DNA copy number and RNA expression data from patient #160.** Copy number plot with the x-axis denoting each point across the genome - each chromosome is highlighted in a different colour starting with chromosome 1 on the left side. Y-axis enumerates the log-2 copy number value at each point across the genome. Red arrows indicate gene amplifications highlighted in this manuscript and corresponding RNASeq expression readouts are displayed in blue boxes. Normal *E2F3* copy number in primary tumour (log_2_ copy number ratio 0.07) and amplification of *E2F3* in the metastasis (log_2_ copy number ratio 1.17). Normal *CDK4* copy number in primary tumour (log_2_ copy number ratio 0.20) and amplification of *CDK4* in the metastasis (log_2_ copy number ratio 1.53). Normal *AKT1* copy number in primary tumour (log_2_ copy number ratio 0.16) and amplification of *AKT1* in the metastasis (log_2_ copy number ratio 1.12).

Focused analysis of 32 selected genes by Iyer et al. described the prevalence and co-occurrence of potentially actionable alterations in a group of 21 genes from signaling pathways known to be relevant in primary UC bladder [5]. We compared the frequency of amplifications and deletions between primary tumors and metastases in these 21 genes as well as another 11 regions found to have statistically significant focal SCNAs in the TCGA analysis (Table 2) [3-7,9]. A log_2_ ratio cut-off of +/− 0.8 was used to stringently define gene amplification and deletion, respectively, based on prior published studies as described in the methods section [7,18,19]. Overall, there were more amplifications/deletions in these genes in metastases compared with primary tumors (8.6% loci altered vs. 4.5%, p < 0.001 Fishers exact, Table 2). In an individual gene-wise comparison, there were more *E2F3* amplifications in metastases compared with primary tumours (30% vs. 7%, p = 0.041 Fishers exact, Table 2 and Figure 1). In 2 of the 11 patients with matched primary and metastatic tissue, *E2F3* amplifications were present in the metastasis but not in the matched primary tumor. In these cases, there was associated increased *E2F3* RNA expression in the metastasis compared with the matched primary (patients 25 and 169, Figures 2 and 3). The *SOX4* locus is located close to *E2F3*, is co-amplified in many of these cases, and was identified by TCGA investigators as another potentially biologically relevant gene in this amplicon [3]. Indeed in these two patients (25 and 169) there was a 2-fold increased RNA expression of *SOX4* between the primary and metastasis specimens that paralleled the gene amplification.

**Table 2:** **Frequency of amplifications and deletions in a focused analysis of 32 genomic regions which were either previously known to be of interest in urothelial cancer or which were identified by TCGA as having statistically significant focal copy number changes**

Pathway	Gene	% alterations in primaries (n = 29)	% alterations in metastasis (n = 30)
MAP kinase pathway	*ERBB2^*	2/29-7%	4/30-13%
	*FGFR3^*	0/29-0%	1/30-3%
	*FGFR1^*	2/29-7%	4/30-13%
	*EGFR^*	0/29-0%	0/30-0%
	*MET^*	0/29-0%	0/30-0%
	*KRAS^*	0/29-0%	0/30-0%
	*NF1^*	0/29-0%	1/30-3%
	*BRAF^*	0/29-0%	0/30-0%
	*RAF1^*	1/29-3%	3/30-10%
	*MYC^*	0/29-0%	2/30-7%
	*MYCL1^*	5/29-17%	3/30-10%
P53 pathway	*MDM2^*	1/29-3%	3/30-10%
	*TP53* ^*∨*^	2/29-7%	2/30-7%
RB pathway*	*CDKN2A* ^*∨*^	8/29-28%	10/30-33%
	*CDK4* *^*	1/29-3%	2/30-7%
	*CCND1* *^*	2/29-7%	6/30-20%
	*CCNE1^*	2/29-7%	4/30-13%
	*RB1* ^*∨*^	0/29-0%	2/30-7%
	E2F3^ *(p = 0.04)	2/29-7%	9/30-30%
PI3K pathway	*PTEN* ^*∨*^	1/29-3%	1/30-3%
	*PIK3CA^*	0/29-0%	0/30-0%
	*AKT1^*	0/29-0%	1/30-3%
	*TSC1* ^*∨*^	1/29-3%	1/30-3%
	*MTOR^*	0/29-0%	0/30-0%
Others	*BCL2L1^*	1/29-3%	1/30-3%
	*PPARG^*	1/29-3%	5/30-17%
	*CREBBP* ^*∨*^	0/29-0%	1/30-3%
	*PVRL4^*	2/29-7%	7/30-23%
	*YWHAZ^*	5/29-17%	4/30-13%
	*NCOR1* ^*∨*^	2/29-7%	1/30-3%
	*YAP1^*	0/29-0%	1/30-3%
	*ZNF703^*	1/29-3%	3/30-10%
		n = 928	n = 960
% total loci with amplification/deletion*	(p < 0.001)	42/928-4.5%	83/960-8.6%

*p <0.05 Fishers exact test, ^=amplification, ^∨^ = deletion.

The data are represented using a threshold of log_2_ copy number ratio >0.8 for amplification and log_2_ copy number ratio < −0.8 for deletion. Data are shown in tabular format with frequency of amplifications and deletions of genes outlined. ^denotes amplifications and ˇdenotes deletions.

### Instances of discordant genetic alterations between paired primary and metastatic samples

**Figure 4:**
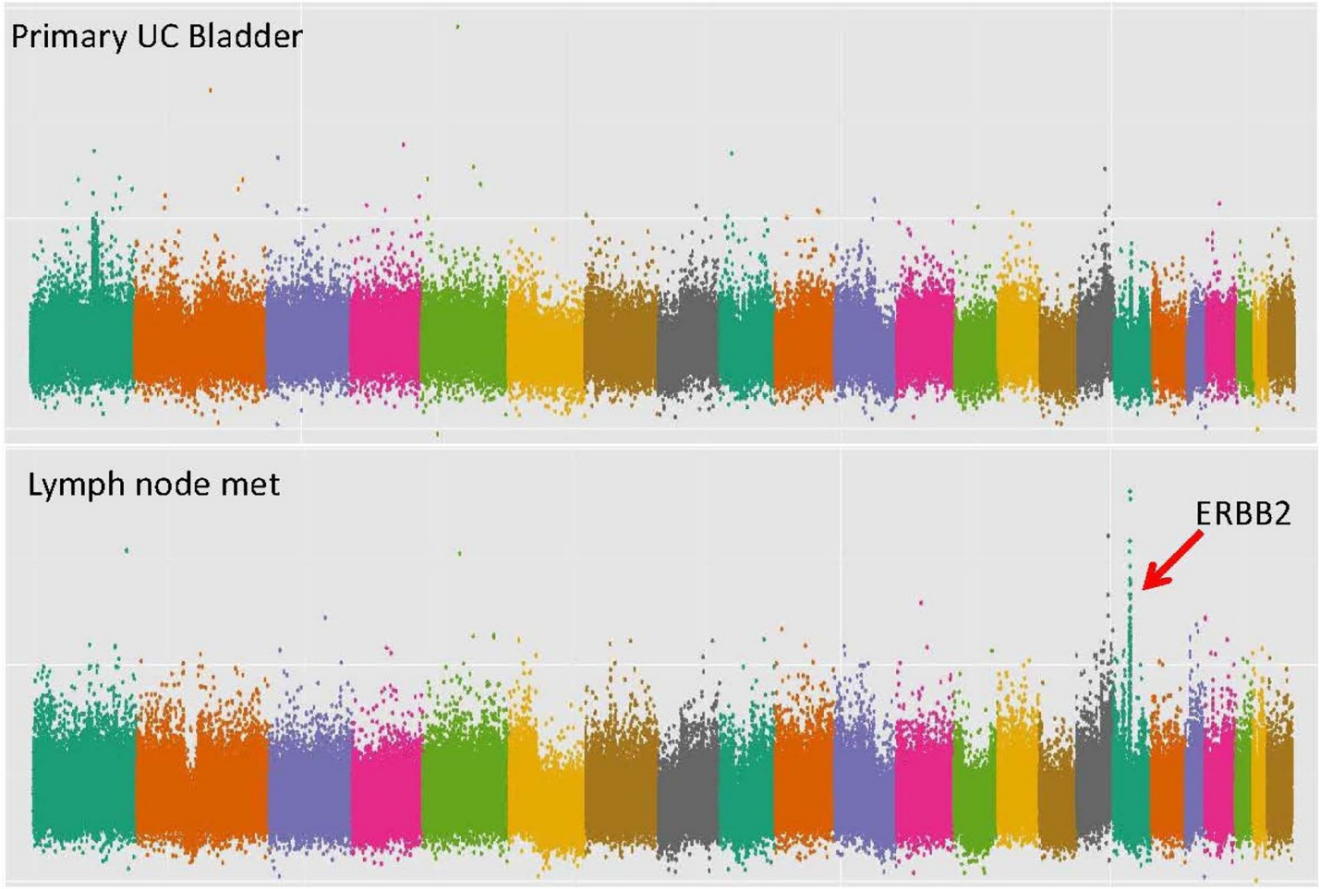
**DNA copy number data from patient #63.** Copy number plot with the x-axis denoting each point across the genome - each chromosome is highlighted in a different colour starting with chromosome 1 on the left side. Y-axis enumerates the log-2 copy number value at each point across the genome. Red arrow indicates gene amplification highlighted in this manuscript. Normal *ERRB2* copy number in primary tumour (log_2_ copy number ratio 0.34) and amplification of *E2F3* in the metastasis (log_2_ copy number ratio 1.19).

In 3 of 11 patients for whom primary and metastatic tissue was available, potentially clinically actionable amplifications were observed in metastases but not in the matched primary tumours (Figures 2, 3 and 4). In the first case (patient 25), *E2F3* and *CCND1* amplifications were detected in a soft tissue renal metastasis and were not present in the corresponding bladder primary (Figure 2). RNA expression data was concordant with these findings with 7.0-fold and 10.6-fold increased expression levels of *E2F3* and *CCND1* respectively. The second case (patient 160) had amplifications of *E2F3*, *CDK4* and *AKT1* in a lung metastasis which were not present in the bladder primary (Figure 3). RNA expression data confirmed increased *E2F3* and *AKT1* expression but not increased *CDK4* expression. The third case (patient 63) had *ERBB2* ampli fication in a lymph node metastasis that was not present in the corresponding bladder primary (Figure 4). RNA expression data was not available for this patient. When examining these 32 genes, we did not find any instance of amplification or deletion in the primary tumor that was not present in a matched metastasis.

### Frequency of low-amplitude copy number alterations

**Figure 5:**
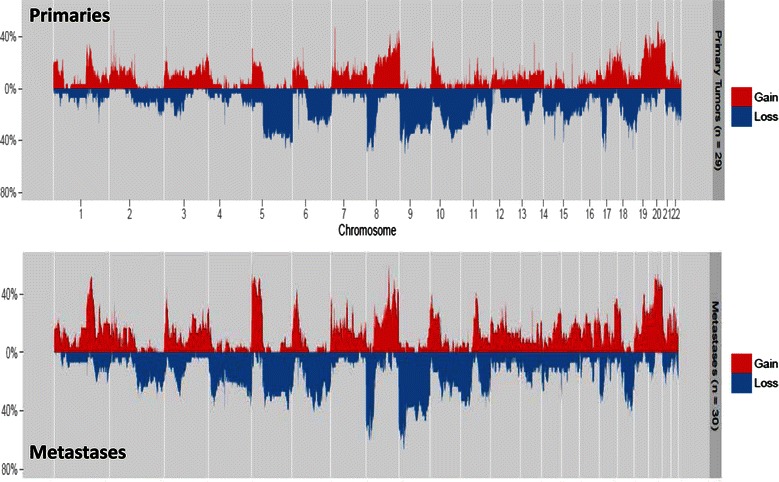
**Low amplitude copy number alterations in primary vs metastatic tumors.** Copy number frequency plots displaying the frequency of copy number gain (CNG) and copy number loss (CNL) at different points across the genome using a cut-off log_2_ ratio +/− 0.25 for CNG and CNL, respectively. The x axis represents the different chromosomes and the y-axis quantifies the percentage of samples with copy number loss or gain greater than the +/− 0.25 log_2_ ratio cut-off.

The frequency of low-amplitude SCNAs across the whole genome was compared between primary and metastatic tumors. A log_2_ ratio cut-off of +/− 0.25 was used to define low-amplitude SCNAs as described in the methods section. Of note, the limited sample size meant our power to detect significant differences after correcting for multiple testing was only 0.4. On a genome-wide basis, the overall fraction of altered loci was not significantly different between primary tumors and metastases. There was a trend towards more chromosome 4 CNLs in metastases compared with primary tumors (Figure 5), although the difference was not statistically significant after correction for multiple testing (p = 0.01 for chromosome 4q and p = 0.03 for chromosome 4p - paired t-test; FDR = 0.31).The trend to more frequent chromosome 4 CNL events in metastases was also observed when the analysis was restricted to paired primary and metastatic tissue specimens from the same patients (p = 0.04 for Chr 4q and p = 0.09 for 4p - paired t-test) ,suggesting that a proportion of tumors may lose genetic material from chromosome 4 when progressing from primary to metastatic disease (Additional file 4: Figure S3). Whether this loss represents a driver or passenger event is unclear.

### GISTIC 2.0

**Figure 6:**
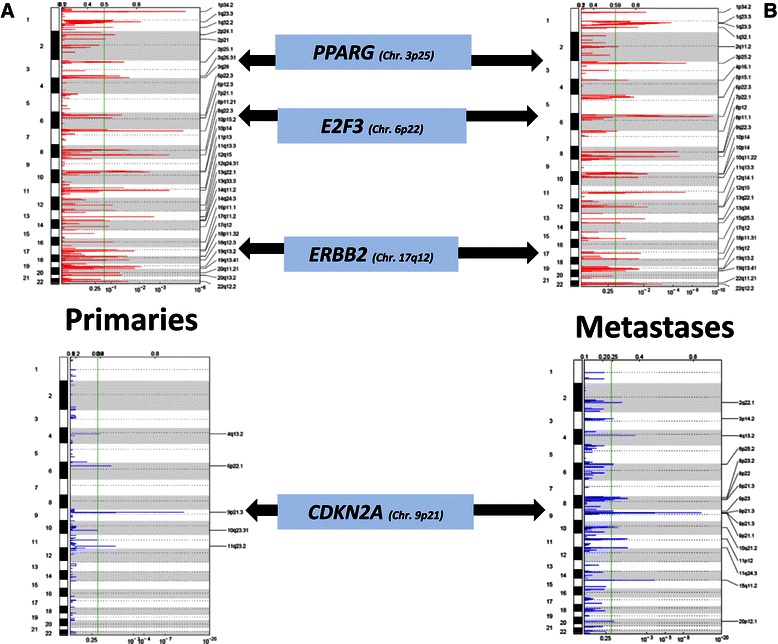
**GISTIC 2.0 analysis of primary (a) and metastatic (b) cohorts.** Copy number data was analysed using GISTIC 2.0 as described in the methods section. The y-axis represents the chromosomal location and the x-axis quantifies the q-value at that point in the genome. The green line denotes cut-off q-value of 0.25 which was used to determine significant events. Red peaks refer to amplifications and blue peaks to deletions.

GISTIC 2.0 analysis of the primary and metastatic cohorts (designed to identify significant driver SCNAs) demonstrated multiple regions of significant SCNA as previously described in other cohorts of UC patients [3,5,7,8,21]. These include regions of amplification at *E2F3*, *ERBB2* and *PPARG* and deletion at *CDKN2A* in both primary (Figure 6a) and metastatic (Figure 6b) cohorts. A recent study, using primary tumour tissue for analysis, reported that amplification at the 1q23.3 locus was associated with worse prognosis in metastatic UC [25]. In GISTIC 2.0 analysis of our dataset, 1q23.3 was found to be significantly amplified in both primary and metastatic samples.

### Hierarchical clustering analysis

**Figure 7:**
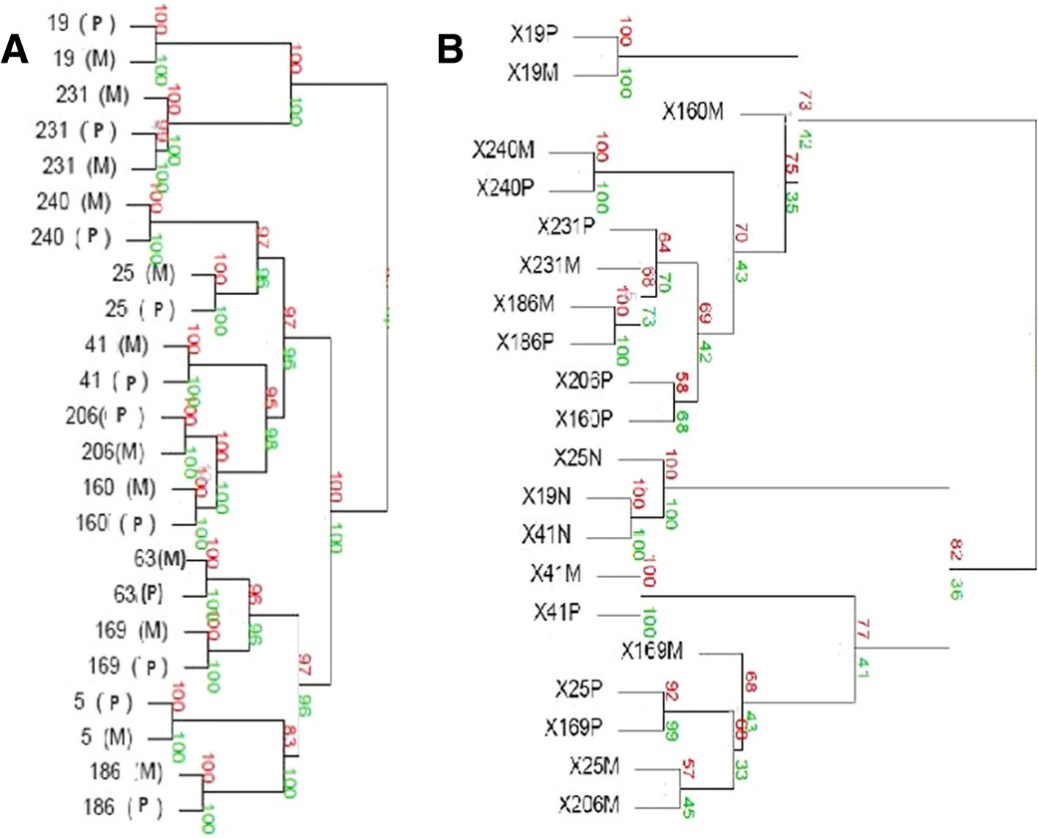
**Hierarchical clustering analysis.** Hierarchical clustering analysis using both DNA **(a)** and RNA **(b)** datasets. The bootstrapping procedure estimates how strongly the clusters are supported by data. Bootstrap values are estimated using multi-scale bootstrap resampling (shown in red numbers) and normal resampling (shown in green numbers), and are reported as percentages, indicating how often a cluster was observed in the bootstrapping [34]. P = primary tumor, M = metastasis, N-normal tissue. For the RNA clustering plot, P = primary tumor, M = metastasis, N = normal tissue. Note: for patient 231 two separate brain metastases were analyzed and both clustered together as shown.

Hierarchical cluster analysis using DNA copy number data confirmed that the paired primary and metastatic samples from the same patients cluster together in all cases suggesting clonality (i.e. that they had initially arisen from a single cell of origin) (Figure 7a). These findings were further corroborated using the MSKCC clonality tool which suggested a high likelihood that 10 of the 11 paired primary and metastatic tumors had arisen from the same cell of origin rather than from different primary tumors. For one patient (patient 169), there was a weak and statistically non-significant trend towards independence. (Additional file 5: Figure S4); however, the primary and metastasis samples from this patient clustered together on hierarchical clustering analysis, suggesting clonality (Figure 7a).

Hierarchical clustering analysis using the RNA expression data from normal and tumor tissue found that the normal tissue specimens clustered together and independent of primary and metastatic tumor samples (Figure 7b). 7 of 9 matched primary and metastatic pairs clustered together and for those that did not (patients 160 and 206) the bootstrap values were poor, suggesting that the high confidence pairings (i.e. those with bootstrap values closer to 100%) are indeed clonal and that RNA expression profiles globally are maintained between the paired primary and metastatic tumors.

## Discussion

These data provide the first comprehensive assessment of SCNAs in metastatic UC. Amplification or deletion of genes involved in the RB signalling pathway were seen in 60% (18 of 30) of metastases, which is of interest given the significant activity of CDK4 inhibitors in other cancers [26,27]. The data also show a higher frequency of *E2F3* amplifications in metastases compared with primary UC and concordant increased *E2F3* RNA expression in patients with *E2F3* amplifications. Prior data from primary UC and other cancers has suggested *E2F3* amplification is associated with higher grade and stage primary tumours [7,28]. Whether E2F3 activity is a functional driver of metastatic progression or simply a marker for more aggressive disease is not yet clear. Iyer et al. recently showed *E2F3* amplification is associated with increased expression of several downstream targets in UC suggesting that, when present, this amplification event results in biologic alterations in this disease [5]. The *SOX4* locus, which is located close to *E2F3,* may also be a biologically relevant gene within this amplicon as it is co-amplified in many of these cases as well as having associated increased RNA expression.

Overall, there were more amplifications/deletions in metastases compared with primary tumours. This is in keeping with the longstanding model of cumulative genetic change leading to cancer evolution and progression as originally described by Nowell et al. almost 30 years ago [29]. More recently Li et al. demonstrated the clonal evolution of primary bladder UC as illustrated by single cell exome analysis from multiple parts of the same tumor [30]. Of note, there were some instances of amplification in primary tumours that were not present in metastases (e.g. the AHR gene on chromosome 7 in patient 160, Figure 3b) suggesting a divergent rather than longitudinal pattern of evolution whereby different clones can form a branched evolutionary tree despite all arising from a common ancestral cell. This is also in keeping with prior data in this disease [31].

In 3 of 11 patients for whom primary and metastatic tissue was available, there were amplifications in metastases that were not present in the primary tumors, including at the *ERBB2*, *AKT1, CDK4, CCND1* and *E2F3* loci. Accompanying total RNA sequencing was available in 2 patients and showed corresponding increased expression levels in several of these genes. This discordance between paired primary and metastatic tissue may have clinical relevance in the era of genomic medicine since the genetic information gleaned from analysing primary tumors may not represent the relevant drivers in metastatic disease. For example, if genomic information from the primary tumour was used to inform therapeutic decision-making for patients 63 and 160 (Figures 3 and 4), the *AKT1* and *ERBB2* amplifications would not have been evident and these patients would not have been considered for HER2 or AKT-mTOR pathway directed therapies. Studies in colon and lung cancer have found similar instances of discordant SCNAs in cancer-related genes when comparing paired primary and metastatic tissue from the same patients [32,33]. On the other hand, these studies reported high rates of concordance (>90%) when examining clinically actionable somatic point mutations (including mutations in *EGFR* and *KRAS*). The discordance in potentially actionable alterations noted in the data presented here suggest that rates of discordance may differ on a gene-by-gene basis and that discordance in SCNAs may be more common than in somatic point mutations.

One important limitation of the data is the relatively small number of samples analysed which limited the power of the study.

## Conclusions

These data can be used to provide an overview of the SCNA landscape in metastatic UC. The intrapatient genomic discrepancies found between primary and metastatic tumours highlights the potential limitations in using archival primary tumour tissue to guide targeted therapy for metastatic disease. Increased frequency of *E2F3* amplification in metastases points to the relevance of the RB pathway in UC with potential therapeutic implications given the ongoing development of multiple CDK inhibitors.

## Additional files

Additional file 1: Table S1. Sites of tumour and normal tissue used for both DNA and RNA extraction. For each of the 46 patients analysed, the age, gender, tissue site for primary, metastatic and normal control samples are outlined. Data not entered denotes that no specimen was available for that patient. All patients had tumor DNA analysis performed. *denotes patients for which tumor RNA analysis was also performed.
Additional file 2: Figure S1. Comparative marker analysis of differentially expressed genes between normal and tumor tissue. The results of a comparative marker analysis of differentially expressed genes represented as a heat map. Each column denotes a single sample, and each differentially expressed gene is represented in an individual row. Only genes that are differentially expressed in normal bladder (sample number indicated followed by the letter “N”) compared to primaries (“P”) and metastases (“M”) are displayed. This analysis was performed with 1000 permutations. Red shading indicates higher relative expression and blue shading indicates lower relative expression. In addition to the gene name, a relative rank of the comparative over- or under-expression as well as a p-value and False Discovery Rate (FDR)-corrected and Bonferroni-correct p-value are given. The relative fold-change between the aggregate expression in the normal samples versus tumors (primary and metastases) is also presented.
Additional file 3: Figure S2. Comparative marker analysis of differentially expressed genes between primary and metastatic tumor tissue. The results of a comparative marker analysis of differentially expressed genes are presented as a heat map. Each sample is represented in a column and each differentially expressed gene is represented in an individual row. Only genes that are differentially expressed in primaries (“P”) compared to metastases (“M”) are shown. This analysis was performed with 1000 permutations. Red shading indicates higher relative expression and blue shading indicates lower relative expression. In addition to the gene name, a relative rank of the comparative over- or under-expression as well as a p-value and False Discovery Rate (FDR)-corrected and Bonferroni-correct p-value are given. The relative fold-change between the aggregate expression in the primary tumors and metastases is also displayed.
Additional file 4: Figure S3. Low amplitude copy number alterations in matched pairs of primary vs metastatic tumors. Copy number frequency plots for the 11 patients with available matched primary and metastatic tissue. The plots display the frequency of copy number gain (CNG) and copy number loss (CNL) at different points across the genome using a cut-off log_2_ ratio +/− 0.25 for CNG and CNL, respectively. The x axis represents the different chromosomes and the y-axis quantifies the percentage of samples with copy number loss or gain greater than the +/− 0.25 log_2_ ratio cut-off.
Additional file 5: Figure S4. MSKCC clonality tool analysis. Genomic copy-number profiles for all 11 primary tumor/metastasis pairs. These plot visualize copy numbers (log base 2 ratios, y-axis) for all segments along the genome (x-axis). Shown odds ratios are calculated using the Clonality R package. This package provides implementations of statistical tests to determine whether two samples from the same patient are independent or clonal.
