# Supplementary material for: DNA copy number analysis of metastatic urothelial carcinoma with comparison to primary tumors
Source: BMC Cancer. 2015 Apr 9;15:242. doi: 10.1186/s12885-015-1192-2 (PMC4392457; doi:10.1186/s12885-015-1192-2)
Supplement: Additional file 1: Table S1. — Sites of tumour and normal tissue used for both DNA and RNA extraction. For each of the 46 patients analysed, the age, gender, tissue site for primary, metastatic and normal control samples are outlined. Data not entered denotes that no specimen was available for that patient. All patients had tumor DNA analysis performed. *denotes patients for which tumor RNA analysis was also performed. [file 12885_2015_1192_MOESM1_ESM.pdf]

Patient ID	Gender	Age	Primary sample	Histology	Primary T stage	Metastatic sample	Normal control
5	Female	52	Bladder	High grade UC (micropapillary differentiation)	T3	Lung	Pelvic node
19*	Male	75	Bladder	High grade UC	T3	Pelvic node	Pelvic node
25*	Female	72	Bladder	High grade UC	T3	Kidney	Pelvic node
41*	Male	61	Bladder	High grade UC (squamous differentiation)	T3	Peritoneum	Pelvic node
63	Female	52	Bladder	High grade UC	T4	Pelvic node	Pelvic node
160*	Male	72	Bladder	High grade UC	T3	Lung	Seminal vesicle
169*	Female	58	Ureter	High grade UC	T3	Pelvic mass	Pelvic node
186*	Female	64	Ureter	High grade UC	T1	Lung	Kidney
206*	Male	48	Bladder	UC carcinoma in situ	Tis	Pelvic node	Pelvic node
231*	Female	51	Ureter	High grade UC	T3	Brain x 2	Kidney
240*	Male	70	Ureter	High grade UC	T2	Lung	Kidney
10	Male	54	Bladder	High grade UC	T2		
29	Male	65	Bladder	High grade UC	T3		Pelvic node
31	Male	61	Bladder	High grade UC	T3		Pelvic node
34	Male	69	Bladder	High grade UC	T3		Pelvic node
60	Female	35	Bladder	High grade UC	T1		Pelvic node
62	Male	68	Bladder	High grade UC	T2		Pelvic node
65	Male	64	Bladder	High grade UC	T2		Seminal vesicle
81	Male	63	Bladder	High grade UC	T1		Pelvic node
82	Male	73	Bladder	High grade UC	T2		Pelvic node
95	Male	63	Bladder	High grade UC	T2		Pelvic node
104	Female	64	Bladder	High grade UC	T1		Pelvic node
110	Male	57	Bladder	High grade UC	T1		Pelvic node
116	Male	59	Bladder	UC carcinoma in situ	Tis		Pelvic node
121	Female	82	Bladder	High grade UC	T2		Pelvic node
181	Male	56	Bladder	High grade UC	T2		Pelvic node
182	Male	72	Bladder	High grade UC	T4		Seminal vesicle
216	Male	68	Ureter	High grade UC	T3		Perinephric node
239	Male	55	Ureter	High grade UC	T3		Renal parenchyma
64	Male	71				Lung	Lung
150	Male	66				Peritoneum	Pelvic node
159	Female	51				Lung	
176	Female	60				Perineum	
187	Male	71				Peritoneum	Abdominal node
188	Male	68				Pelvic node	Prostate
195	Female	69				Pelvic node	
198	Male	73				Peritoneum	
203	Female	64				Lung	
207	Male	52				Peritonuem	
211	Female	45				Brain	
215	Female	51				Brain	Pelvic node
218	Female	50				Pericardium	
219	Male	64				Abdominal node	
222	Male	57				Lung	Lung
227	Female	55				Peritoneum	Pelvic node
250	Female	60				Lung	Lung
255	Male	81				Lung	Lung
