# Supplementary figures and images for: DNA copy number analysis of metastatic urothelial carcinoma with comparison to primary tumors
Source: BMC Cancer. 2015 Apr 9;15:242. doi: 10.1186/s12885-015-1192-2 (PMC4392457; doi:10.1186/s12885-015-1192-2)

19N, 25N, 41N vs. 160M, 160P, 169M, 169P, 186M, 186P, 19M, 19P, 206M, 206P, 231M, 231P, 240M, 240P, 25M, 25P, 41M, 41P

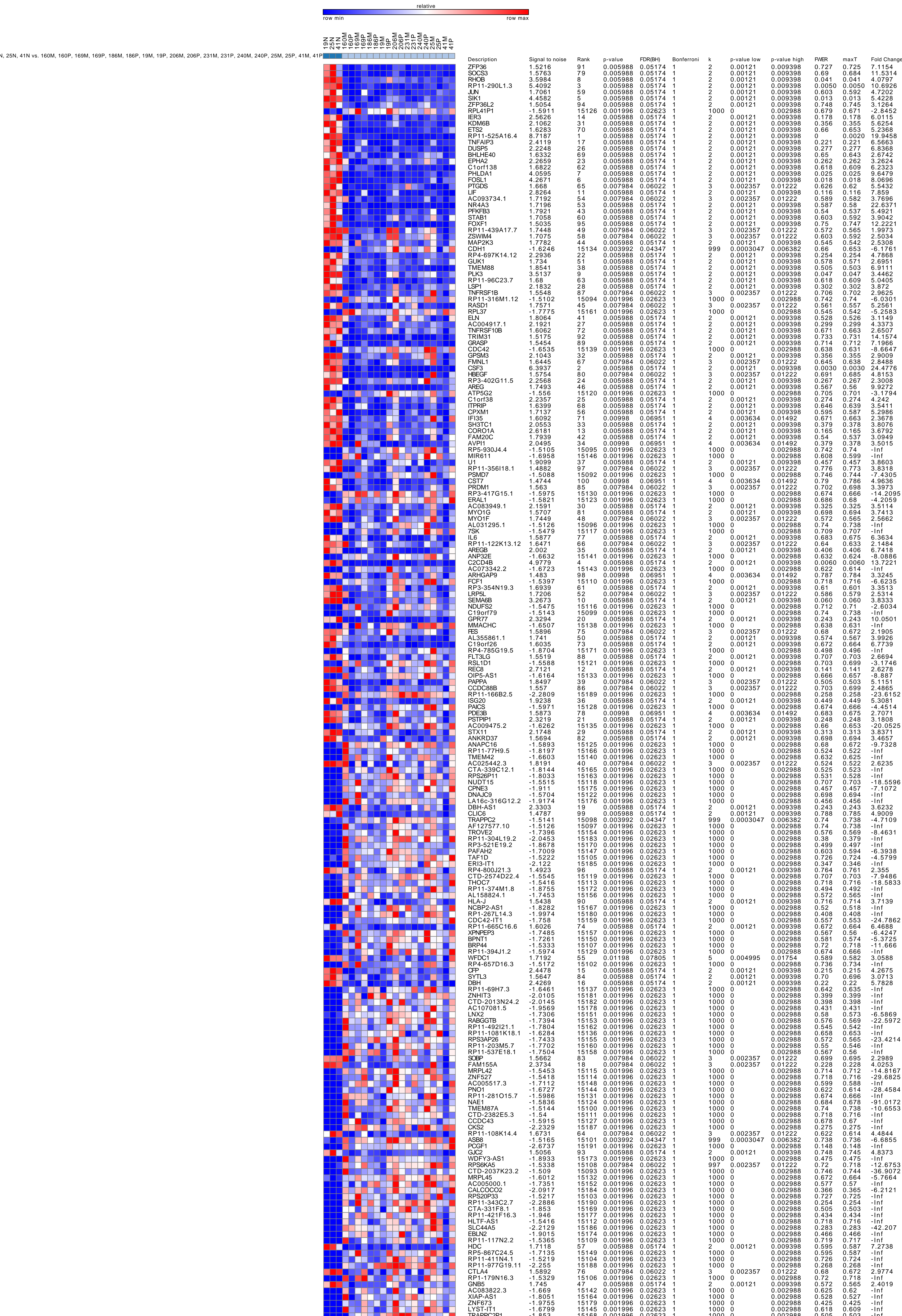

Supplement: Additional file 2: Figure S1. — Comparative marker analysis of differentially expressed genes between normal and tumor tissue. The results of a comparative marker analysis of differentially expressed genes represented as a heat map. Each column denotes a single sample, and each differentially expressed gene is represented in an individual row. Only genes that are differentially expressed in normal bladder (sample number indicated followed by the letter “N”) compared to primaries (“P”) and metastases (“M”) are displayed. This analysis was performed with 1000 permutations. Red shading indicates higher relative expression and blue shading indicates lower relative expression. In addition to the gene name, a relative rank of the comparative over- or under-expression as well as a p-value and False Discovery Rate (FDR)-corrected and Bonferroni-correct p-value are given. The relative fold-change between the aggregate expression in the normal samples versus tumors (primary and metastases) is also presented. [file 12885_2015_1192_MOESM2_ESM.pdf]

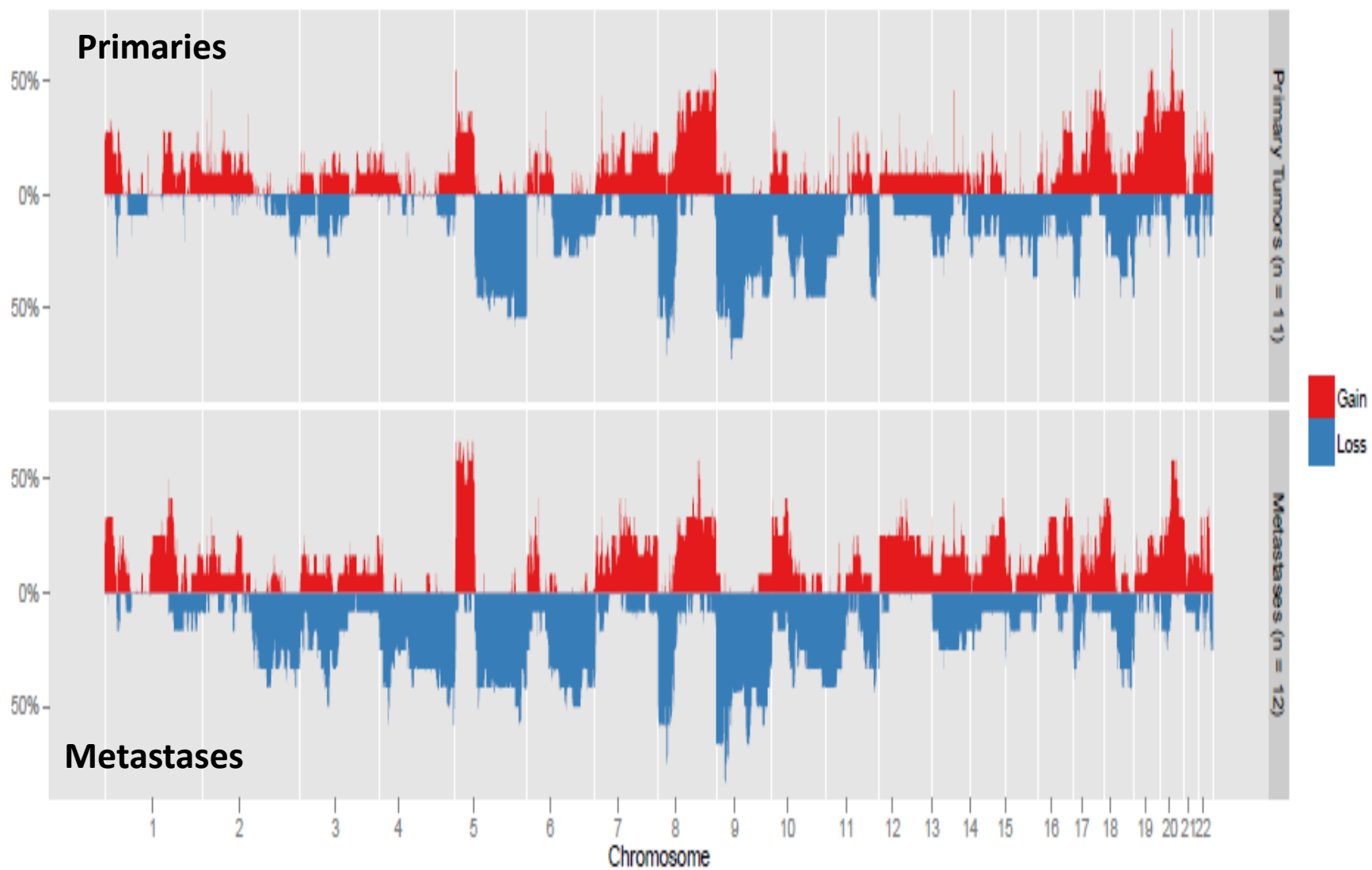

Supplement: Additional file 4: Figure S3. — Low amplitude copy number alterations in matched pairs of primary vs metastatic tumors. Copy number frequency plots for the 11 patients with available matched primary and metastatic tissue. The plots display the frequency of copy number gain (CNG) and copy number loss (CNL) at different points across the genome using a cut-off log2 ratio +/− 0.25 for CNG and CNL, respectively. The x axis represents the different chromosomes and the y-axis quantifies the percentage of samples with copy number loss or gain greater than the +/− 0.25 log2 ratio cut-off. [file 12885_2015_1192_MOESM4_ESM.pdf]

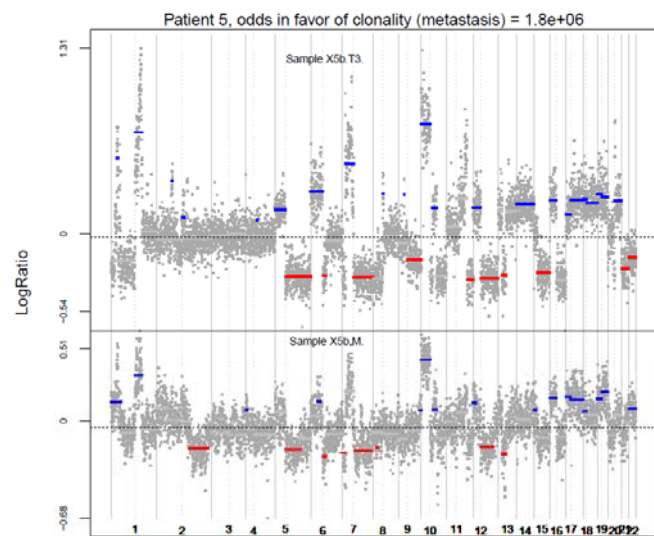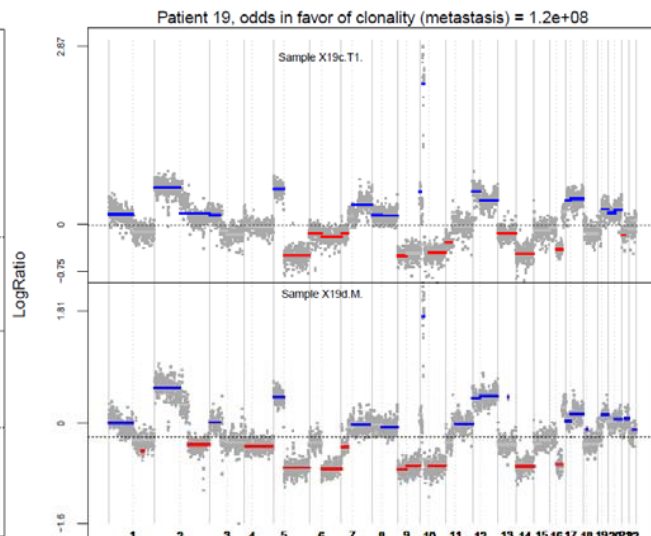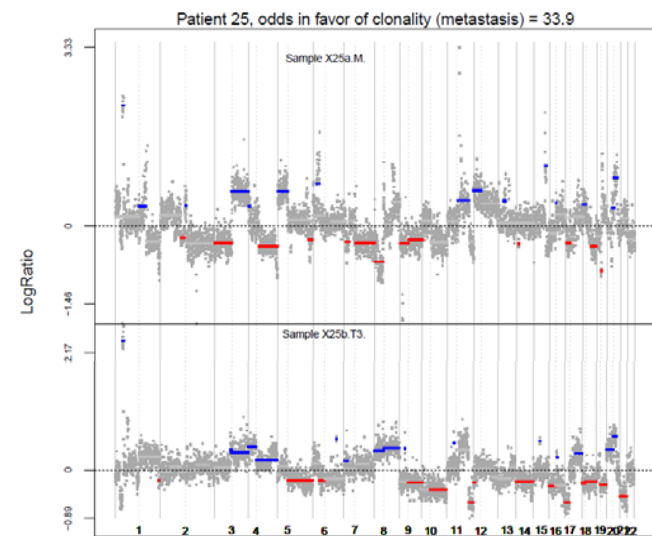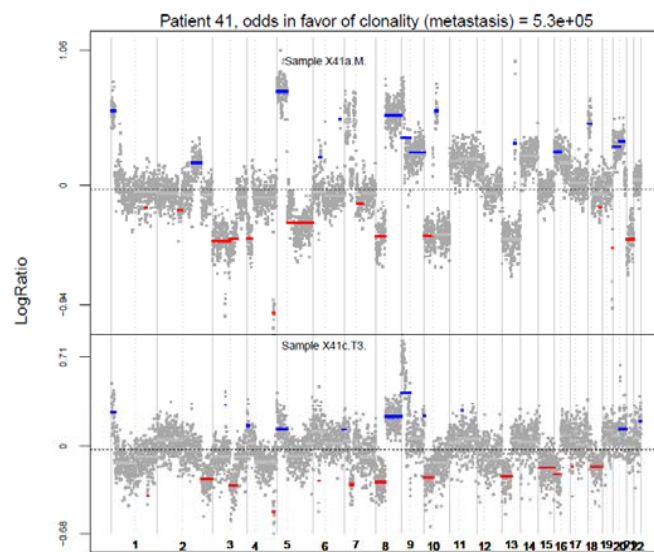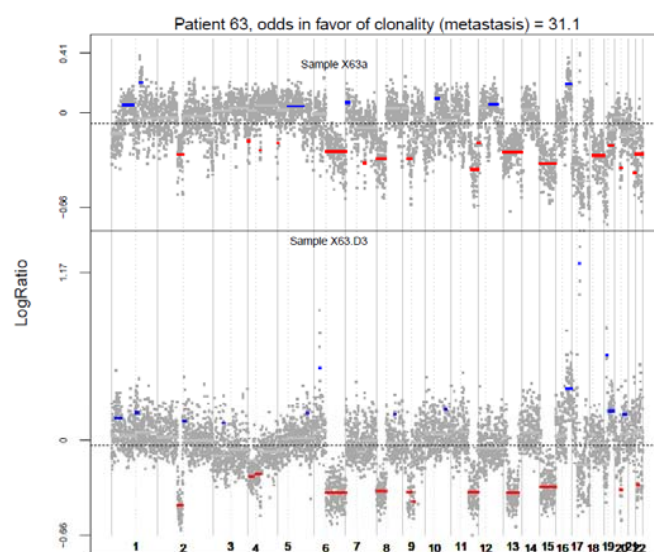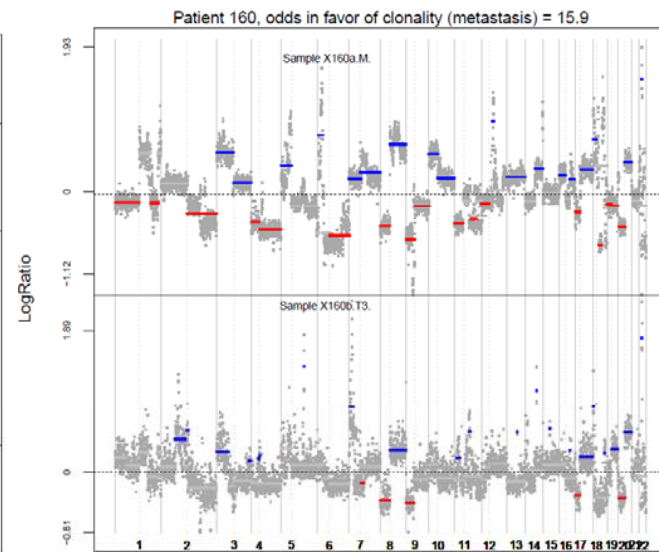

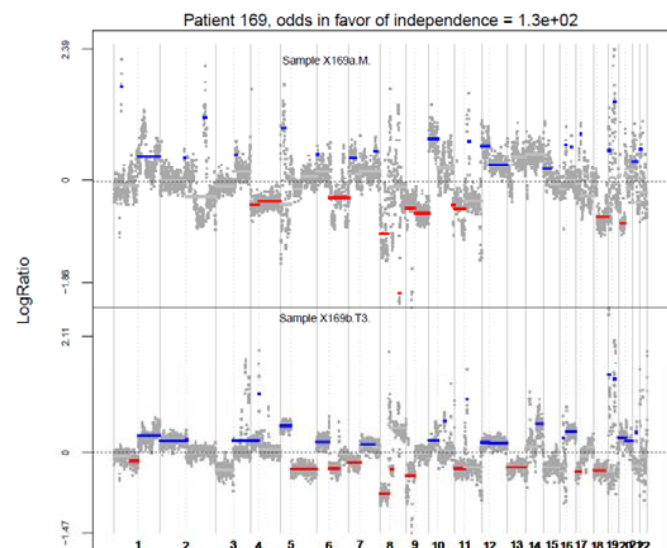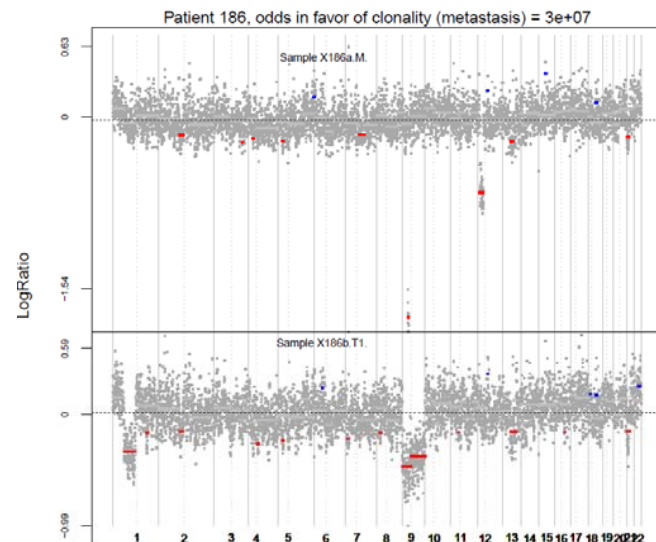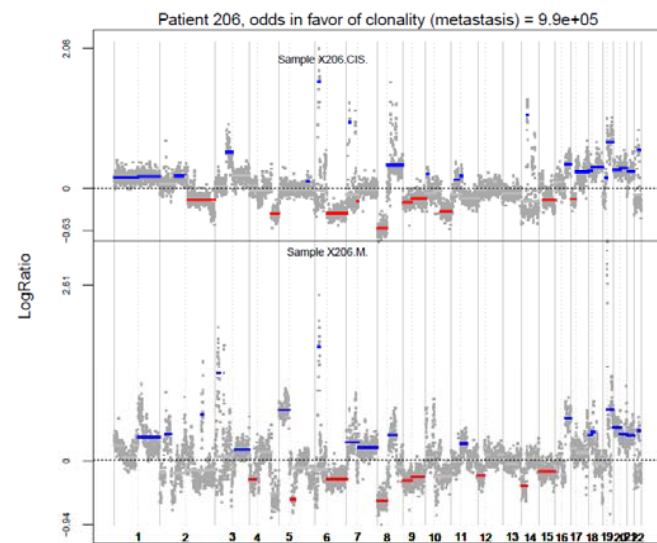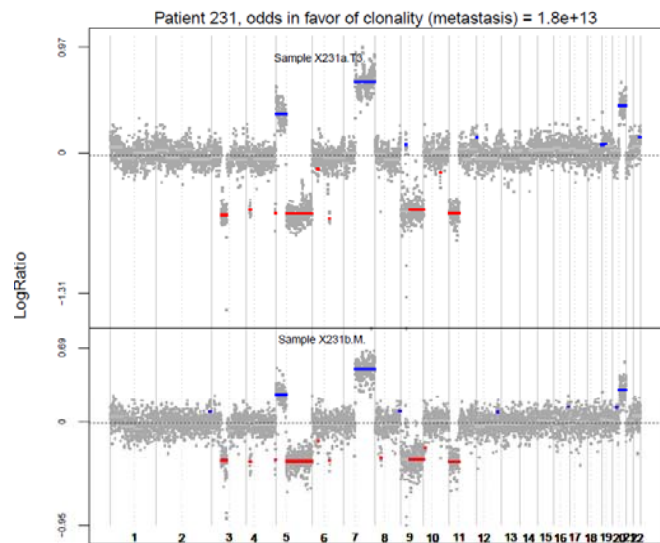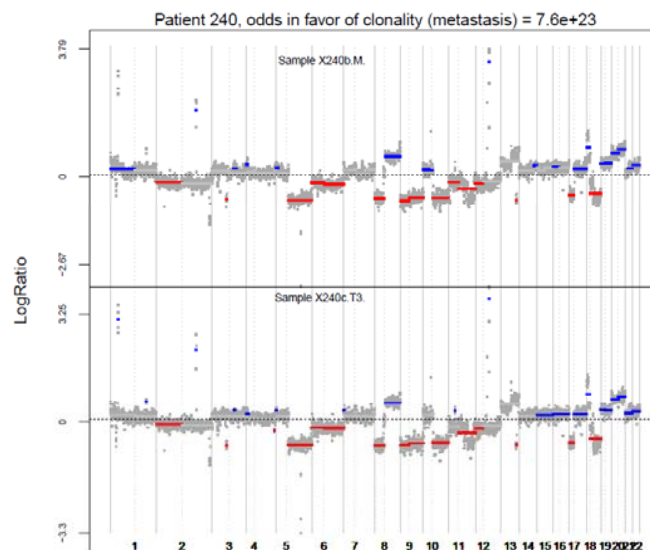

Supplement: Additional file 5: Figure S4. — MSKCC clonality tool analysis. Genomic copy-number profiles for all 11 primary tumor/metastasis pairs. These plot visualize copy numbers (log base 2 ratios, y-axis) for all segments along the genome (x-axis). Shown odds ratios are calculated using the Clonality R package. This package provides implementations of statistical tests to determine whether two samples from the same patient are independent or clonal. [file 12885_2015_1192_MOESM5_ESM.pdf]
