# Supplementary material for: DNA copy number analysis of metastatic urothelial carcinoma with comparison to primary tumors
Source: BMC Cancer. 2015 Apr 9;15:242. doi: 10.1186/s12885-015-1192-2 (PMC4392457; doi:10.1186/s12885-015-1192-2)
Supplement: Additional file 3: Figure S2. — Comparative marker analysis of differentially expressed genes between primary and metastatic tumor tissue. The results of a comparative marker analysis of differentially expressed genes are presented as a heat map. Each sample is represented in a column and each differentially expressed gene is represented in an individual row. Only genes that are differentially expressed in primaries (“P”) compared to metastases (“M”) are shown. This analysis was performed with 1000 permutations. Red shading indicates higher relative expression and blue shading indicates lower relative expression. In addition to the gene name, a relative rank of the comparative over- or under-expression as well as a p-value and False Discovery Rate (FDR)-corrected and Bonferroni-correct p-value are given. The relative fold-change between the aggregate expression in the primary tumors and metastases is also displayed. [file 12885_2015_1192_MOESM3_ESM.pdf]

160P, 169P, 186P, 19P, 206P, 231P, 240P, 25P, 41P vs. 160M, 169M, 186M, 19M, 206M, 231M, 240M, 25M, 4

A

B

160P, 169P, 186P, 19P, 206P, 231P, 240P, 25P, 41P vs. 160M, 169M, 186M, 19M, 206M, 231M, 240M, 25M, 41M

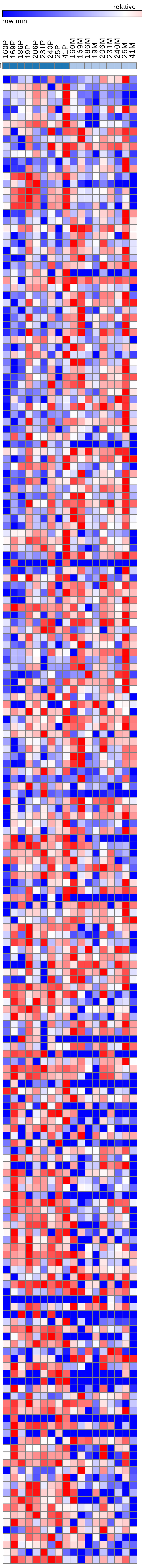

Description	Signal to noise	Rank	p-value	FDR(BH)	Bonferroni	k	p-value low	p-value high	FWER	maxT	Fold Change
FTH1	-0.5177	15151	0.06188	0.9994	1	970	0.04639	0.07586	1	1	-1.1072
DES	0.8594	11	0.003992	0.9994	1	1	0.0003047	0.006382	0.992	0.992	3.0612
MYH11	0.9507	5	0.005988	0.9994	1	2	0.00121	0.009398	0.945	0.943	0.991
AF001548.5	0.6919	50	0.01597	0.9994	1	7	0.007887	0.02262	1	1	2.1324
PKM2	-0.5029	15143	0.06387	0.9994	1	969	0.04816	0.07808	1	1	-1.322
CNN1	0.9091	8	0.003992	0.9994	1	1	0.0003047	0.006382	0.971	0.969	105.0642
AF001548.6	0.9401	6	0.007984	0.9994	1	3	0.002357	0.01222	0.948	0.946	8.7883
ALDOA	-0.4828	15130	0.06786	0.9994	1	967	0.05171	0.08251	1	1	-1.2529
PLD3	-0.4752	15121	0.07186	0.9994	1	965	0.05528	0.08692	1	1	-1.3884
RP11-67L3.5	-0.5511	15166	0.0519	0.9994	1	975	0.03762	0.06468	1	1	-11.8634
ACTG2	0.7016	46	0.005988	0.9994	1	2	0.00121	0.009398	1	1	-5.3519
CSNK2B	-0.4571	15098	0.07385	0.9994	1	964	0.05707	0.08912	1	1	-1.8506
TUBA1B	-0.5175	15150	0.05389	0.9994	1	974	0.03937	0.06693	1	1	-1.432
RN5S5	0.911	7	0.001996	0.9994	1	0	0	0.002988	0.971	0.969	3.099
TAC3	0.7275	35	0.003992	0.9994	1	1	0.0003047	0.006382	1	1	-1.4293
RN5S13	0.6065	97	0.01796	0.9994	1	8	0.009393	0.0251	1	1	2.3442
RN5S3	0.6156	88	0.01397	0.9994	1	6	0.006418	0.0201	1	1	1.9699
RN5S10	0.8122	15	0.003992	0.9994	1	1	0.0003047	0.006382	0.999	0.999	3.044
RP11-386G11.10	-0.4803	15127	0.08184	0.9994	1	960	0.06426	0.09789	1	1	-1.5086
MYBL2	-0.4815	15128	0.07784	0.9994	1	962	0.06066	0.09351	1	1	0.5463
INF2	-0.4725	15119	0.07186	0.9994	1	965	0.05528	0.08692	1	1	-3.717
HIST1H4D	-0.6647	15185	0.01796	0.9994	1	992	0.009393	0.0251	1	1	-1.4821
LPCAT1	-0.5352	15163	0.04591	0.9994	1	978	0.03243	0.05791	1	1	1.8756
NCOR2	-0.4646	15109	0.08583	0.9994	1	958	0.06787	0.1023	1	1	-1.6998
MAZ	-0.4526	15094	0.09381	0.9994	1	954	0.07513	0.111	1	1	-1.4181
TTYH3	-0.5336	15162	0.0479	0.9994	1	977	0.03415	0.06017	1	1	-1.84
AC053503.6	1.0354	3	0.005988	0.9994	1	2	0.00121	0.009398	0.826	0.819	-2.5555
RP11-67L3.6	-0.4992	15140	0.03393	0.9994	1	984	0.02225	0.04415	1	1	0.3797
LMOD1	0.6787	57	0.01597	0.9994	1	7	0.007887	0.02262	1	1	-3.8833
RP11-388M20.6	-0.5564	15169	0.05589	0.9994	1	973	0.04112	0.06917	1	1	-2.2372
H2AFX	-0.6804	15188	0.02196	0.9994	1	990	0.01249	0.02997	1	1	-4.0166
GNA12	-0.4755	15123	0.07186	0.9994	1	965	0.05528	0.08692	1	1	0.6368
SDHA	-0.4974	15135	0.05988	0.9994	1	971	0.04463	0.07363	1	1	2.8941
FOXM1	-0.468	15112	0.09182	0.9994	1	955	0.07331	0.1088	1	1	0.04804
CLPTM1L	-0.5194	15154	0.05589	0.9994	1	973	0.04112	0.06917	1	1	-108.4015
SYNPO2	0.7812	20	0.005988	0.9994	1	2	0.00121	0.009398	1	1	-3.4691
IMPDH1	-0.5244	15156	0.06986	0.9994	1	966	0.05349	0.08472	1	1	0.1867
ADAM33	0.6123	92	0.02196	0.9994	1	10	0.01249	0.02997	1	1	-1.7867
MCM5	-0.4824	15129	0.08383	0.9994	1	959	0.06606	0.1001	1	1	0.7872
TYMS	-0.7263	15191	0.01198	0.9994	1	995	0.004995	0.01754	1	1	0.02765
ARL2	-0.5648	15172	0.04591	0.9994	1	978	0.03243	0.05791	1	1	52.3603
RNF126	-0.5117	15147	0.05389	0.9994	1	974	0.03937	0.06693	1	1	-1.8402
MAPK1IP1L	-0.4699	15116	0.07585	0.9994	1	963	0.05886	0.09132	1	1	-3.0877
CCL18	-0.5061	15146	0.05988	0.9994	1	971	0.04463	0.07363	1	1	2.4316
PDCD6	-0.5916	15177	0.03593	0.9994	1	983	0.02392	0.04646	1	1	4.266
TCF19	-0.6392	15182	0.01397	0.9994	1	994	0.006418	0.0201	1	1	2.3569
TUBG1	-0.4757	15124	0.07585	0.9994	1	963	0.05886	0.09132	1	1	-5.4758
CD276	-0.4512	15092	0.09182	0.9994	1	955	0.07331	0.1088	1	1	0.6051
CD99P1	0.6162	87	0.02595	0.9994	1	12	0.01568	0.03476	1	1	-1.2244
FXYD2	0.6273	84	0.01198	0.9994	1	5	0.004995	0.01754	1	1	-1.5724
ABCA3	-0.4532	15095	0.07585	0.9994	1	963	0.05886	0.09132	1	1	2.0169
YIF1A	-0.5042	15144	0.06387	0.9994	1	969	0.04816	0.07808	1	1	3.0684
CACNA1H	0.8198	13	0.005988	0.9994	1	2	0.00121	0.009398	0.999	0.999	-1.8981
CCT5	-0.5202	15155	0.06387	0.9994	1	969	0.04816	0.07808	1	1	0.06987
HIST1H2AB	-0.4596	15102	0.08583	0.9994	1	958	0.06787	0.1023	1	1	3.7447
SLC11A1	-0.5723	15174	0.03593	0.9994	1	983	0.02392	0.04646	1	1	2.5459
AC009133.14	-0.5376	15164	0.05988	0.9994	1	971	0.04463	0.07363	1	1	11.439
AKR7A2	-0.5535	15168	0.04591	0.9994	1	978	0.03243	0.05791	1	1	0.2095
CLN6	-0.471	15117	0.09182	0.9994	1	955	0.07331	0.1088	1	1	4.5387
PGP	-0.5012	15141	0.06387	0.9994	1	969	0.04816	0.07808	1	1	9.4441
SPNS1	-0.465	15110	0.07585	0.9994	1	963	0.05886	0.09132	1	1	2.597
PGM5	0.7513	29	0.01397	0.9994	1	6	0.006418	0.0201	1	1	-1.8
MRGPRF	0.6772	60	0.01397	0.9994	1	6	0.006418	0.0201	1	1	-2.0031
MATN2	0.6705	63	0.01397	0.9994	1	6	0.006418	0.0201	1	1	-1.5637
TOX4	-0.6528	15184	0.01597	0.9994	1	993	0.007887	0.02262	1	1	0.3717
AC015849.1	0.663	70	0.007984	0.9994	1	3	0.002357	0.01222	1	1	-1.2238
STARD10	-0.5049	15145	0.05589	0.9994	1	973	0.04112	0.06917	1	1	5.3937
CHRDL2	0.7684	25	0.01198	0.9994	1	5	0.004995	0.01754	1	1	-2.2962
NUTF2	-0.4676	15111	0.07984	0.9994	1	961	0.06246	0.0957	1	1	0.7133
SLC35E1	-0.5255	15157	0.05589	0.9994	1	973	0.04112	0.06917	1	1	1.4661
RP11-334E6.10	-0.6252	15180	0.02595	0.9994	1	988	0.01568	0.03476	1	1	0.1403
7SK	0.6992	47	0.00998	0.9994	1	4	0.003634	0.01492	1	1	-1.5912
SSSCA1	-0.5148	15149	0.05389	0.9994	1	974	0.03937	0.06693	1	1	3.2812
SLC29A1	-0.5769	15175	0.02595	0.9994	1	988	0.01568	0.03476	1	1	9.3136
RP11-494K3.2	0.7274	36	0.01796	0.9994	1	8	0.009393	0.0251	1	1	-1.7818
NDUFS8	-0.4932	15134	0.05988	0.9994	1	971	0.04463	0.07363	1	1	0.223
TRIB3	-0.4562	15097	0.08982	0.9994	1	956	0.0715	0.1066	1	1	1.3688
NOP10	-0.4644	15108	0.07784	0.9994	1	962	0.06066	0.09351	1	1	3.1131
LMNB1	-0.4735	15120	0.09581	0.9994	1	953	0.07695	0.1131	1	1	1.9535
CDT1	-0.4991	15139	0.06387	0.9994	1	969	0.04816	0.07808	1	1	2.4725
MARCO	-0.6937	15189	0.01597	0.9994	1	993	0.007887	0.02262	1	1	1.7409
MRPS18A	-0.4987	15138	0.07585	0.9994	1	963	0.05886	0.09132	1	1	143.4066
MAP2K1	-0.7101	15190	0.01796	0.9994	1	992	0.009393	0.0251	1	1	5.0301
RGS5	0.7099	43	0.00998	0.9994	1	4	0.003634	0.01492	1	1	-2.3386
SNORA70G	-0.4573	15099	0.08184	0.9994	1	960	0.06426	0.09789	1	1	1.5599
COL14A1	0.6112	94	0.01996	0.9994	1	9	0.01093	0.02755	1	1	-1.8227
RGP1	-0.5139	15148	0.05988	0.9994	1	971	0.04463	0.07363	1	1	2.5352
FADS3	-0.4801	15126	0.05988	0.9994	1	971	0.04463	0.07363	1	1	2.2321
TCF21	0.805	17	0.01198	0.9994	1	5	0.004995	0.01754	1	1	-1.7397
CTD-2196E14.9	-0.5631	15171	0.03393	0.9994	1	984	0.02225	0.04415	1	1	2.4023
MTX1P1	-0.5184	15153	0.05589	0.9994	1	973	0.04112	0.06917	1	1	1.614
PKMYT1	-0.5618	15170	0.05988	0.9994	1	971	0.04463	0.07363	1	1	1.8416
THOC6	-0.4752	15122	0.08782	0.9994	1	957	0.06968	0.1044	1	1	2.2577
NFASC	0.6578	74	0.01397	0.9994	1	6	0.006418	0.0201	1	1	-1.7553
C12orf32	-0.4687	15115	0.1078	0.9994	1	947	0.08794	0.1261	1	1	1.4836
BASP1P1	0.689	51	0.00998	0.9994	1	4	0.003634	0.01492	1	1	-1.9293
CDH16	0.6834	55	0.00998	0.9994	1	4	0.003634	0.01492	1	1	-1.3845
AP000350.3	-0.5819	15176	0.03194	0.9994	1	985	0.02059	0.04182	1	1	1.6835
STXBP1	-0.4606	15105	0.07385	0.9994	1	964	0.05707	0.08912	1	1	1.7362
LSM12P1	-0.4921	15133	0.05589	0.9994	1	973	0.04112	0.06917	1	1	1.6974
TNNT2	0.7249	38	0.00998	0.9994	1	4	0.003634	0.01492	1	1	-1.5331
CDCA5	-0.4574	15100	0.0998	0.9994	1	951	0.08061	0.1174	1	1	1.6604
IGHV3-72	0.9614	4	0.003992	0.9994	1	1	0.0003047	0.006382	0.941	0.939	-1.6032
AL355861.1	0.661	72	0.00998	0.9994	1	4	0.003634	0.01492	1	1	-1.5635
PAMR1	0.6366	81	0.01796	0.9994	1	8	0.009393	0.0251	1	1	-1.4203
LA16c-366D3.1	0.6684	64	0.01996	0.9994	1	9	0.01093	0.02755	1	1	-1.3917
RP11-1260E13.1	-0.4575	15101	0.07585	0.9994	1	963	0.05886	0.09132	1	1	1.286
RASL11A	0.6064	98	0.03593	0.9994	1	17	0.02392	0.04646	1	1	-1.5899
ASF1B	-0.4831	15131	0.07385	0.9994	1	964	0.05707	0.08912	1	1	1.4937
PQLC2	-0.5292	15160	0.05389	0.9994	1	974	0.03937	0.06693	1	1	1.8277
RN5S202	1.2502	1	0.003992	0.9994	1	1	0.0003047	0.006382	0.424	0.427	-1.8043
AL365332.1	-0.6208	15179	0.02196	0.9994	1	990	0.01249	0.02997	1	1	1.9979
CHAF1B	-0.5183	15152	0.02994	0.9994	1	986	0.01894	0.03948	1	1	1.4112
AIMP2	-0.6761	15187	0.01198	0.9994	1	995	0.004995	0.01754	1	1	2.0422
AC018738.2	0.6051	100	0.02794	0.9994	1	13	0.0173	0.03713	1	1	-1.8927
DACT1	0.6101	96	0.03393	0.9994	1	16	0.02225	0.04415	1	1	-1.5983
AC004945.1	0.6978	48	0.01198	0.9994	1	5	0.004995	0.01754	1	1	-1.3643
ACE	-0.4555	15096	0.09182	0.9994	1	955	0.07331	0.1088	1	1	1.489
RP11-83B20.1	-0.4632	15107	0.07585	0.9994	1	963	0.05886	0.09132	1	1	1.51
HDDC3	-0.529	15159	0.0479	0.9994	1	977	0.03415	0.06017	1	1	1.8018
AL139423.1	-0.4686	15113	0.08982	0.9994	1	956	0.0715	0.1066	1	1	1.5685
MLST8	-0.5378	15165	0.05988	0.9994	1	971	0.04463	0.07363	1	1	1.7279
ARMCX2	0.6566	75	0.02196	0.9994	1	10	0.01249	0.02997	1	1	-1.729
BGLAP	0.6923	49	0.01796	0.9994	1	8	0.009393	0.0251	1	1	-1.6045
KB-1562D12.1	1.0765	2	0.001996	0.9994	1	0	0	0.002988	0.746	0.739	-1.7151
RNU5A-1	0.6061	99	0.02595	0.9994	1	12	0.01568	0.03476	1	1	-1.7802
RP11-753H16.5	0.6226	86	0.02595	0.9994	1	12	0.01568	0.03476	1	1	-1.4237
PAQR4	-0.652	15183	0.01996	0.9994	1	991	0.01093	0.02755	1	1	1.4263
CDCA8	-0.4796	15125	0.08583	0.9994	1	958	0.06787	0.1023	1	1	1.5652
MIR1181	0.8106	16	0.00998	0.9994	1	4	0.003634	0.01492	1	1	-1.3974
TRIP13	-0.4974	15136	0.08184	0.9994	1	960	0.06426	0.09789	1	1	1.4844
J01415.16	0.6123	93	0.03194	0.9994	1	15	0.02059	0.04182	1	1	-1.6667
CENPM	-0.639	15181	0.02196	0.9994	1	990	0.01249	0.02997	1	1	1.4405
AC003989.4	0.7167	41	0.005988	0.9994	1	2	0.00121	0.009398	1	1	-1.7124
FMR1-IT1	0.6559	76	0.01796	0.9994	1	8	0.009393	0.0251	1	1	-1.6436
LCE1F	0.8029	18	0.03593	0.9994	1	17	0.02392	0.04646	1	1	-1.3772
PLA2G3	0.8576	12	0.003992	0.9994	1	1	0.0003047	0.006382	0.992	0.992	-1.3135
ACSS2	-0.4713	15118	0.07585	0.9994	1	963	0.05886	0.09132	1	1	1.4743
AMELX	0.6135	91	0.02595	0.9994	1	12	0.01568	0.03476	1	1	-1.2797
RP3-461P17.10	0.6106	95	0.02595	0.9994	1	12	0.01568	0.03476	1	1	-1.4217
RNF152	0.7435	31	0.007984	0.9994	1	3	0.002357	0.01222	1	1	-1.5716
CTD-2501E16.2	0.7826	19	0.005988	0.9994	1	2	0.00121	0.009398	1	1	-1.4703
CTD-2589H19.4	-0.5017	15142	0.07385	0.9994	1	964	0.05707	0.08912	1	1	1.4693
CSN3	0.6144	89	0.03393	0.9994	1	16	0.02225	0.04415	1	1	-1.3357
DGUOK	-0.4631	15106	0.08583	0.9994	1	958	0.06787	0.1023	1	1	1.3663
AKT3-IT1	0.707	44	0.01397	0.9994	1	6	0.006418	0.0201	1	1	-1.5525
RP11-239H6.2	-0.6171	15178	0.02595	0.9994	1	988	0.01568	0.03476	1	1	1.6922
AC007875.3	0.6888	52	0.02395	0.9994	1	11	0.01408	0.03238	1	1	-1.6503
WFDC1	0.6659	67	0.01597	0.9994	1	7	0.007887	0.02262	1	1	-1.4064
RPS16P5	0.8169	14	0.007984	0.9994	1	3	0.002357	0.01222	0.999	0.999	-1.703
AC098680.4	0.7535	28	0.02994	0.9994	1	14	0.01894	0.03948	1	1	-1.475
RP11-395G23.3	0.7711	24	0.005988	0.9994	1	2	0.00121	0.009398	1	1	-1.5905
RTP4	0.6641	68	0.02196	0.9994	1	10	0.01249	0.02997	1	1	-1.4998
DACT3	0.6321	82	0.02994	0.9994	1	14	0.01894	0.03948	1	1	-1.3828
AC012072.1	0.6138	90	0.02595	0.9994	1	12	0.01568	0.03476	1	1	-1.6897
HAND2	0.6841	54	0.02196	0.9994	1	10	0.01249	0.02997	1	1	-1.4487
RP11-347C18.4	0.6275	83	0.005988	0.9994	1	2	0.00121	0.009398	1	1	-1.6402
AC104135.2	0.7211	39	0.01397	0.9994	1	6	0.006418	0.0201	1	1	-1.5101
CTB-193M12.3	0.7048	45	0.007984	0.9994	1	3	0.002357	0.01222	1	1	-1.5984
RP11-379F4.1	0.6721	62	0.00998	0.9994	1	4	0.003634	0.01492	1	1	-1.3839
RP11-275F13.1	-0.4891	15132	0.05589	0.9994	1	973	0.04112	0.06917	1	1	1.3001
RPP38	-0.4687	15114	0.06587	0.9994	1	968	0.04994	0.0803	1	1	1.5194
AL136458.1	0.7439	30	0.007984	0.9994	1	3	0.002357	0.01222	1	1	-1.8486
ZNF668	-0.4598	15103	0.09182	0.9994	1	955	0.07331	0.1088	1	1	1.3155
RP11-462G2.1	-0.5702	15173	0.1457	0.9994	1	928	0.1232	0.1667	1	1	1.1669
FUT1	-0.4516	15093	0.06786	0.9994	1	967	0.05171	0.08251	1	1	1.2243
RN5S429	0.6661	66	0.2156	0.9994	1	107	0.1893	0.2401	1	1	-1.1924
LCE6A	0.7631	26	0.003992	0.9994	1	1	0.0003047	0.006382	1	1	-1.5193
RP11-29M5.1	0.7316	33	0.00998	0.9994	1	4	0.003634	0.01492	1	1	-1.7853
AC118754.4	0.7118	42	0.005988	0.9994	1	2	0.00121	0.009398	1	1	-1.3595
C8orf84	0.7779	22	0.01198	0.9994	1	5	0.004995	0.01754	1	1	-1.7012
MSLN	-0.4602	15104	0.0978	0.9994	1	952	0.07878	0.1153	1	1	1.3269
SNORA71	-0.669	15186	0.02395	0.9994	1	989	0.01408	0.03238	1	1	1.826
RP11-267J23.4	0.6822	56	0.01597	0.9994	1	7	0.007887	0.02262	1	1	-1.6102
U4	0.7301	34	0.01996	0.9994	1	9	0.01093	0.02755	1	1	-1.5528
POLR2LP	0.6663	65	0.2136	0.9994	1	106	0.1874	0.238	1	1	-1.1412
AC009336.1	0.6785	59	0.01198	0.9994	1	5	0.004995	0.01754	1	1	-1.6576
FAM86FP	-0.4978	15137	0.07186	0.9994	1	965	0.05528	0.08692	1	1	1.2283
RP4-752I6.1	0.6616	71	0.01597	0.9994	1	7	0.007887	0.02262	1	1	-1.589
HOXA11	0.637	80	0.01397	0.9994	1	6	0.006418	0.0201	1	1	-1.3362
RN5S506	0.6609	73	0.2116	0.9994	1	105	0.1855	0.236	1	1	-1.2194
CICP16	0.6258	85	0.01996	0.9994	1	9	0.01093	0.02755	1	1	-1.4495
CTD-2528N2.1	0.9086	9	0.03992	0.9994	1	19	0.0273	0.05107	0.971	0.969	-1.2948
AC009464.1	-0.5293	15161	0.07186	0.9994	1	965	0.05528	0.08692	1	1	1.497
HDC	0.6465	78	0.02595	0.9994	1	12	0.01568	0.03476	1	1	-1.335
CTLA4	0.7546	27	0.00998	0.9994	1	4	0.003634	0.01492	1	1	-1.5667
CBX5P1	0.6881	53	0.003992	0.9994	1	1	0.0003047	0.006382	1	1	-1.4587
RBPMSLP	-0.5511	15167	0.03792	0.9994	1	982	0.02561	0.04877	1	1	1.3841
RP11-38P22.2	0.6425	79	0.01996	0.9994	1	9	0.01093	0.02755	1	1	-1.4932
RP11-565F19.4	0.7796	21	0.005988	0.9994	1	2	0.00121	0.009398	1	1	-1.4467
RP11-61L14.6	0.776	23	0.001996	0.9994	1	0	0	0.002988	1	1	-1.5772
RP11-618I10.4	0.7357	32	0.01198	0.9994	1	5	0.004995	0.01754	1	1	-1.3843
AC093375.1	0.6786	58	0.007984	0.9994	1	3	0.002357	0.01222	1	1	-1.5423
AC104438.1	0.9084	10	0.001996	0.9994	1	0	0	0.002988	0.971	0.969	-1.5278
XCR1	0.6534	77	0.02395	0.9994	1	11	0.01408	0.03238	1	1	-1.3807
RP1-317E23.3	-0.5275	15158	0.05389	0.9994	1	974	0.03937	0.06693	1	1	1.3145
AL135749.1	0.7177	40	0.007984	0.9994	1	3	0.002357	0.01222	1	1	-1.5571
AC009970.1	0.6748	61	0.01597	0.9994	1	7	0.007887	0.02262	1	1	-1.4746
PROM1	0.7249	37	0.00998	0.9994	1	4	0.003634	0.01492	1	1	-1.356
RP11-134E15.2	0.6636	69	0.01597	0.9994	1	7	0.007887	0.02262	1	1	-1.5801

1. Import File

File:	Clipboard

2. Adjust

Ceiling:	
Floor:	0.01
Log:	true
Inverse log:	false
Subtract mean from each row	false
Subtract median from each row	false
Divide each row by standard deviation:	false
Divide each row by median absolute deviation:	false
Subtract mean from each column:	false
Subtract median from each column:	false
Divide each column by standard deviation:	false
Divide each column by median absolute deviation:	false
Quantile normalization:	false
Rank rormalization:	false

3. Marker Selection

Comparison(s):	160P, 169P, 186P, 19P, 206P, 231P, 240P, 25P, 41P vs. 160M, 169M, 186M, 19M, 206M, 231M, 240M, 25M, 4
Permutations:	1000
Markers:	100
Include all columns in result dataset:	false
Data is in log scale (used for fold change calculation):	false
Compute:	Permutation p-values
Test statistic:	Signal to noise
Use median instead of mean:	false
Ensure standard deviation is at least 5% of the class mean/median:	false
Random number seed	779948224
Save permuted scores:	false
Test direction:	Two sided

GENE-E 3.0.196
Built 02/21/2014 01:53 PM
